# Fluoropyrimidine type, patient age, tumour sidedness and mutation status as determinants of benefit in patients with metastatic colorectal cancer treated with EGFR monoclonal antibodies: individual patient data pooled analysis of randomised trials from the ARCAD database

**DOI:** 10.1038/s41416-024-02604-y

**Published:** 2024-02-24

**Authors:** C. S. Karapetis, H. Liu, M. J. Sorich, L. D. Pederson, E. Van Cutsem, T. Maughan, J. Y. Douillard, C. J. O’Callaghan, D. Jonker, C. Bokemeyer, A. Sobrero, C. Cremolini, B. Chibaudel, J. Zalcberg, R. Adams, M. Buyse, M. Peeters, T. Yoshino, A. de Gramont, Q. Shi

**Affiliations:** 1 Flinders Medical Centre, Adelaide, SA Australia; 2 Flinders University, Adelaide, SA Australia; 3 Mayo Clinic, Rochester, NY USA; 4 University Hospitals Gasthuisberg Leuven and University of Leuven, Leuven, Belgium; 5 University of Liverpool, Liverpool, UK; 6 University of Nantes and Integrated Centers of Oncology ICO Rene Gauducheau Cancer Nantes, Nantes, France; 7 Canadian Cancer Trials Group, Kingston, ON Canada; 8 The Ottawa Hospital Research Institute, Ottawa, ON Canada; 9 University Medical Center Hamburg-Eppendorf, Hamburg, Germany; 10 Ospedale San Martino, Genova, Italy; 11 University of Pisa, Pisa, Italy; 12 Franco-British Institute Levallois-Perre, Levallois-Perre, France; 13 Dept of Medical Oncology, Alfred Health and School of Public Health, Monash University, Melbourne, VIC Australia; 14 Velindre Cancer Centre Cardiff University, Cardiff, UK; 15 International Drug Development Institute, Louvain-la-Neuve, Belgium; 16 Antwerp University and Antwerp University Hospital, Antwerp, Belgium; 17 National Cancer Centre Hospital East, Kashiwa, Japan

**Keywords:** Prognostic markers, Colon cancer, Targeted therapies

## Abstract

**Background:**

KRAS mutations in metastatic colorectal cancer (mCRC) are used as predictive biomarkers to select therapy with EGFR monoclonal antibodies (mAbs). Other factors may be significant determinants of benefit.

**Methods:**

Individual patient data from randomised trials with a head-to-head comparison between EGFR mAb versus no EGFR mAb (chemotherapy alone or best supportive care) in mCRC, across all lines of therapy, were pooled. Overall survival (OS) and progression-free survival (PFS) were compared between groups. Treatment effects within the predefined KRAS biomarker subsets were estimated by adjusted hazard ratio (HR_adj_) and 95% confidence interval (CI). EGFR mAb efficacy was measured within the KRAS wild-type subgroup according to BRAF and NRAS mutation status. In both KRAS wild-type and mutant subgroups, additional factors that could impact EGFR mAb efficacy were explored including the type of chemotherapy, line of therapy, age, sex, tumour sidedness and site of metastasis.

**Results:**

5675 patients from 8 studies were included, all with known mCRC KRAS mutation status. OS (HR_adj_ 0.90, 95% CI 0.84–0.98, *p* = 0.01) and PFS benefit (HR_adj_ 0.73, 95% CI 0.68–0.79, *p* < 0.001) from EGFR mAbs was observed in the KRAS wild-type group. PFS benefit was seen in patients treated with fluorouracil (HR_adj_ 0.75, 95% CI 0.68–0.82) but not with capecitabine-containing regimens (HR_adj_ 1.04, 95% CI 0.86–1.26) (*p*_interaction_ = 0.002). Sidedness also interacted with EGFR mAb efficacy, with survival benefit restricted to left-sided disease (*p*_interaction_ = 0.038). PFS benefits differed according to age, with benefits greater in those under 70 (*p*_interaction_ = 0.001). The survival benefit was not demonstrated in those patients with mutations found in the KRAS, NRAS or BRAF genes. The presence of liver metastases interacted with EGFR mAb efficacy in patients with KRAS mutant mCRC (*p*_interaction_ = 0.004).

**Conclusion:**

The benefit provided by EGFR mAbs in KRAS WT mCRC is associated with left-sided primary tumour location, younger patient age and absence of NRAS or BRAF mutations. Survival benefit is observed with fluorouracil but not capecitabine. Exploratory results support further research in KRAS mutant mCRC without liver metastases.

## Introduction

The epidermal growth factor receptor (EGFR) was identified as a potential therapeutic target in the fight against cancer more than 20 years ago [1, 2]. Subsequently, anti-EGFR monoclonal antibodies (mAbs) were developed, particularly cetuximab and panitumumab, and these have changed the treatment landscape for many patients with metastatic colorectal cancer (mCRC) [3]. Improved outcomes with EGFR mAbs have been reported in first, second- and third-line therapy for mCRC [4–8].

Despite the overall benefit, the use of anti-EGFR mAbs in mCRC is associated with treatment-related toxicity and a lack of response in a significant proportion of patients. Moreover, the financial expense of EGFR mAbs is a significant consideration [9]. The cost-effectiveness and therapeutic index of EGFR mAbs have been improved through the identification of molecular biomarkers to predict which patients are more likely to benefit and to determine which patients should not be selected for such treatment. Studies initially demonstrated that benefit derived from the addition of EGFR mAb has limited to patients with tumours wild-type (WT) at KRAS exon 2 and subsequently to “extended RAS” WT tumours, which do not harbour activating mutations at KRAS exons 2,3 and 4 or NRAS exons 2, 3 and 4 [4–6, 8, 10]. Cetuximab and panitumumab were incorporated into international guidelines recommending their use in patients with RAS WT mCRC in conjunction with or after chemotherapy [11, 12].

Some studies, however, have failed to demonstrate survival benefits with the application of EGFR mAbs in patients with KRAS WT mCRC [13, 14]. Inconsistent findings across the trials have been attributed to the nature of the concurrent chemotherapy backbone, patient selection factors and to chance. The true magnitude of the benefit remains unclear. We set out to explore the effect of KRAS mutations on the efficacy of EGFR mAbs in the treatment of mCRC.

## Methods

Individual patient data from randomised trials, collected in the ARCAD database and identified in Project Data Sphere (PDS), with head-to-head comparison between EGFR mAb, administered either alone or with chemotherapy, versus the same treatment without the EGFR mAb (i.e., chemotherapy alone or BSC alone) in mCRC, across all lines of therapy (first, second and later), were pooled. Studies that tested VEGF mAbs only were not considered. For studies that included both EGFR and VEGF mAbs, the treatment arms with VEGF mAbs and a combination of EGFR and VEGF mAbs were excluded. Due to not being adopted in practice, the treatment arms with intermittent use of chemotherapy were also excluded. Only individual mCRC patients with known KRAS mutation status were included in the pooled analysis. Overall survival (OS) and progression-free survival (PFS) were compared within the KRAS WT and KRAS mutant (MT) cohorts by the Cox model, stratified by studies and adjusted by age, gender, and performance status. Treatment effects were estimated by adjusted hazard ratio (HR_adj_) and 95% confidence interval (CI).

The primary study objective was to determine the effect of EGFR mAbs on survival outcomes when used in the treatment of mCRC according to KRAS mutation status. KRAS was selected as the biomarker of interest as this was the first to be reported to be associated with EGFR mAb benefit. The effect of additional biomarkers, principally BRAF and NRAS mutations, particularly within the KRAS WT subgroup, was also explored. We sought to determine if the survival effect of anti-EGFR mAb is influenced by; BRAF and NRAS mutation status, sidedness (left v right), age, sex, type of chemotherapy backbone (oxaliplatin or irinotecan) and type of fluoropyrimdine (fluorouracil or capecitabine). We also evaluated the effect of the line of therapy (first, second or third (last) line). The site of metastatic disease (liver, lung, lymph nodes) and prior surgical interventions (surgery to excise primary, surgery to excise metastases) were also explored. P-values for survival comparisons < 0.01 were considered statistically significant to account for multiple comparisons. Interaction P-values of < 0.05 were considered significant.

## Results

**Fig. 1:**
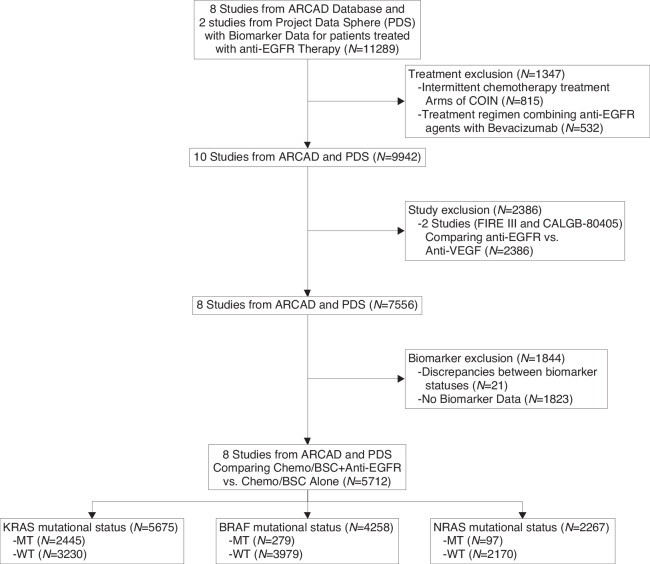
Consort diagram. ARCAD Aide et Recherche en Cancérologie Digestive, EGFR epidermal growth factor receptor, VEGF vascular endothelial growth factor, MT mutant, WT wild-type.

Ten studies from the ARCAD database and PDS included anti-EGFR therapies. Two studies were excluded because they contained bevacizumab in the comparator arm. Among the remaining 8 trials that qualified for inclusion, 815 patients who received intermittent chemotherapy (one treatment arm from the COIN trial) were excluded. An additional 532 patients who received a combination of anti-EGFR agents with bevacizumab were excluded. Furthermore, 1844 patients were excluded where no biomarker data was available. 5675 patients with data available on the KRAS mutation status of the CRC were included and analysed. The consort flow diagram (Fig. 1) details the study and patient selection process.

The included study titles and the number of patients that each study contributed to this analysis are detailed in Supplemental Table 1. Supplemental Table 2 summarises data reported in the original publications of these trials, where only 2 of the 8 included studies demonstrated statistically significant improvement in overall survival in the patients with KRAS WT tumours and none reported benefit in the patients with KRAS MT tumours.

3230 patients had KRAS WT tumours, and 1601 of these patients were treated with EGFR mAbs. 2445 patients had KRAS MT tumours, and 1244 of these patients received EGFR mAbs. Overall survival was prolonged in those patients with KRAS WT mCRC who were treated with an EGFR mAb, but the benefit bordered the predefined significance level (HR_adj_ 0.90, 95% CI 0.84–0.98, *p* = 0.01). PFS was significantly improved with EGFR mAbs in the KRAS WT group (HR_adj_ 0.73, 95% CI 0.68–0.79, *p* < 0.001). Sidedness was a significant determinant of benefit, with EGFR mAb use associated with PFS prolongation in left-side tumours but not right-sided disease (left-sided PFS HR_adj_ 0.74, 95% CI 0.67–i0.83, *p* < 0.001; right-sided PFS HR_adj_ 1.03, 95% CI 0.81–1.32, *p* = 0.798, *p*_interaction_ 0.021).

Where NRAS and BRAF mutation status were known, the presence of mutations in these genes was found to affect the EGFR mAb survival benefit. Patients with KRAS WT mCRC who had NRAS MT did not benefit from EGFR mAbs (OS HR_adj_ 1.52, 95% CI 0.93–2.50, *p* = 0.095, *p*_interaction_ = 0.008; PFS HR_adj_ 1.61, 95% CI 1.00–2.61, *p* = 0.048, *p*_interaction_ = 0.001). The interaction test for overall survival was not significant according to BRAF mutation status (OS HR_adj_ 0.93, 95% CI 0.72–1.20, *p* = 0.566, *p*_interaction_ = 0.775) but it did interact with PFS benefit (PFS HR_adj_ 0.93, 95% CI 0.72–1.21, *p* = 0.608, *p*_interaction_ = 0.014).

When BSC was the control arm (the ‘later line’ trials), EGFR mAb therapy provided OS and PFS benefit in patients with KRAS WT cancers (OS HR_adj_ 0.79, *p* = 0.036 and PFS HR_adj_ 0.41, *p* < 0.001). The PFS benefit observed in these ‘later line’ trials was greater than the PFS benefit seen in the first- and second-line trials (*p*_interaction_ < 0.001) for patients with KRAS WT mCRC.

When fluoropyrimidine-based treatment was the control arm, the choice of fluoropyrimidine made a difference, as the survival benefit was only observed in patients treated with fluorouracil (OS HR_adj_ 0.86, 95% CI 0.79 – 0.95, *p* = 0.003, and PFS HR_adj_ 0.75, 95% CI 0.68–0.82, *p* < 0.001) and not in those treated with capecitabine (OS HR_adj_ 1.09, 95% CI 0.90-1.33 and PFS HR_adj_ 1.04, 95% CI 0.86–1.26). The difference between fluorouracil and capecitabine satisfied the interaction test for OS (*p*_interaction_ = 0.035) and PFS (*p*_interaction_ = 0.002). The benefit associated with EGFR mAb use in patients with KRAS WT mCRC was seen when either irinotecan or oxaliplatin were used in the partnering chemotherapy combination, with no significant difference between the two.

**Fig. 2:**
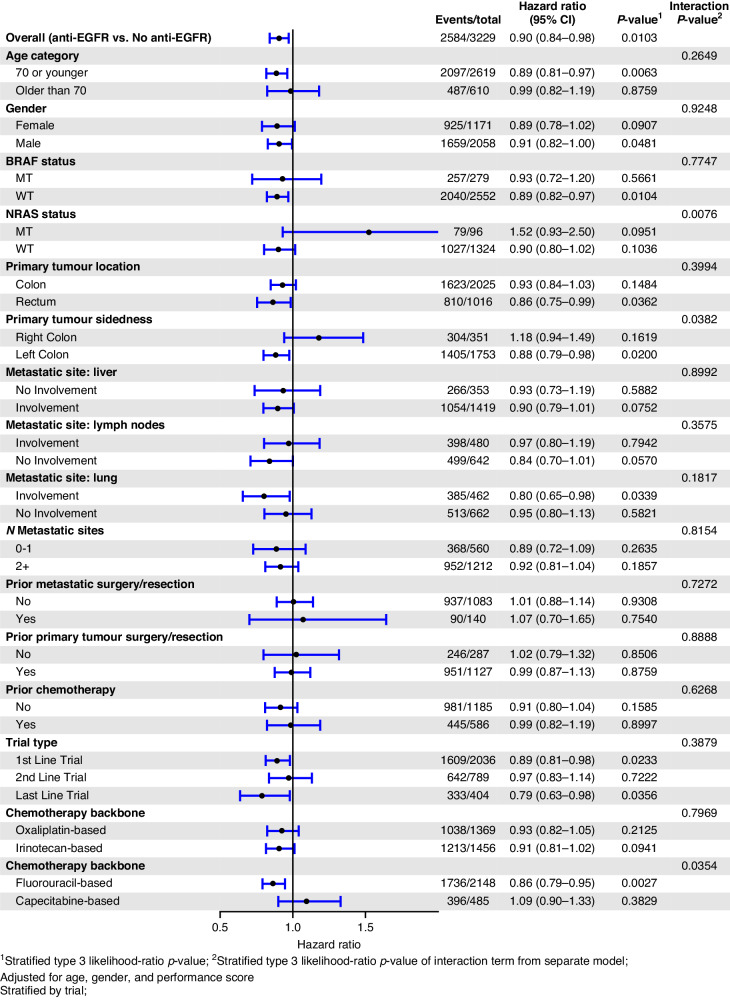
Forest plot of OS in the KRAS wild-type subgroup. Forest plot of overall survival for KRAS wild-type subgroup of patients treated with EGFR mAb + chemo/BSC versus chemo/BSC alone. EGFR epidermal growth factor receptor.

**Fig. 3:**
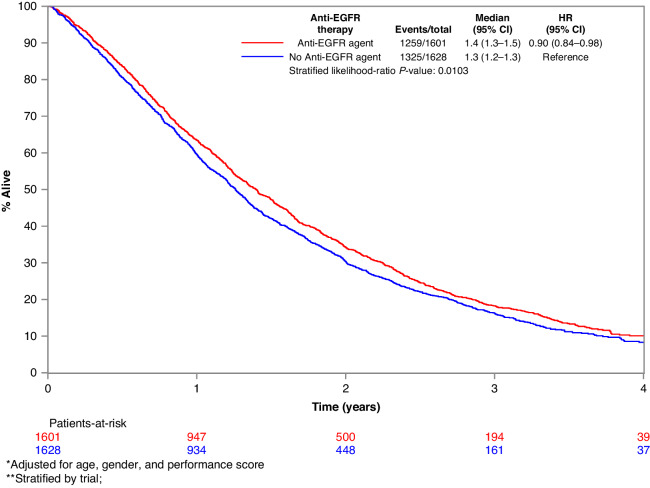
Kaplan–Meier OS curves in the KRAS WT subgroup. Kaplan–Meier overall survival curves for KRAS wild-type subgroup of patients treated with EGFR mAb + chemo/BSC versus chemo/BSC alone. EGFR epidermal growth factor receptor.

**Fig. 4:**
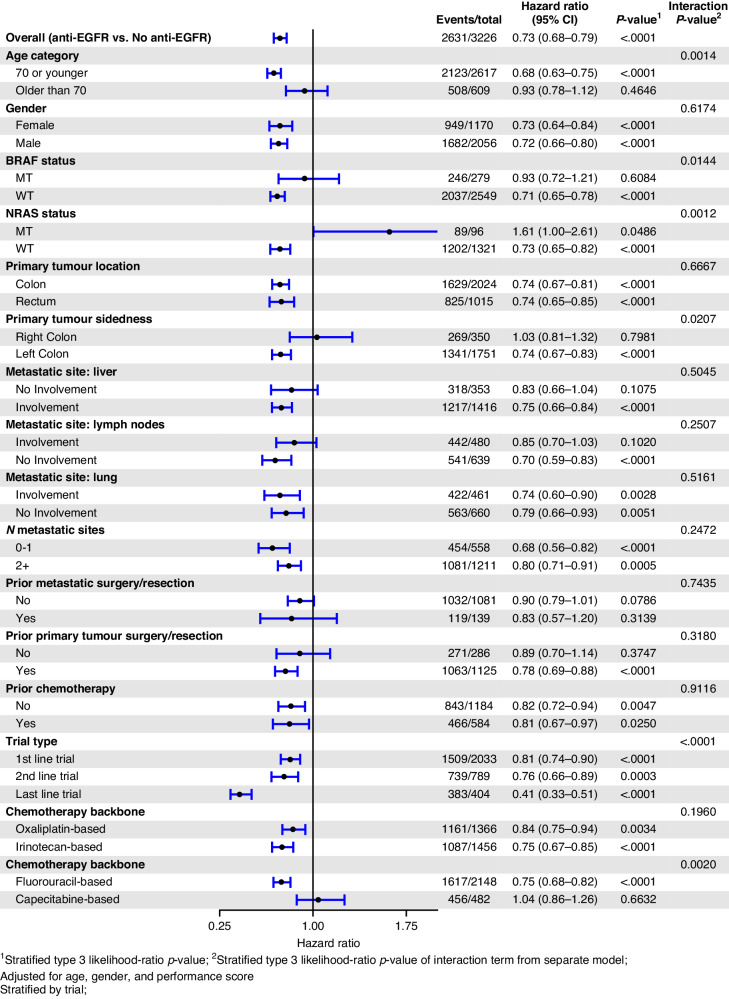
Forest plot—PFS in KRAS wild-type subgroup. Forest plot of progression-free survival for KRAS wild-type subgroup of patients treated with EGFR mAb + chemo/BSC versus chemo/BSC alone. EGFR epidermal growth factor receptor.

**Fig. 5:**
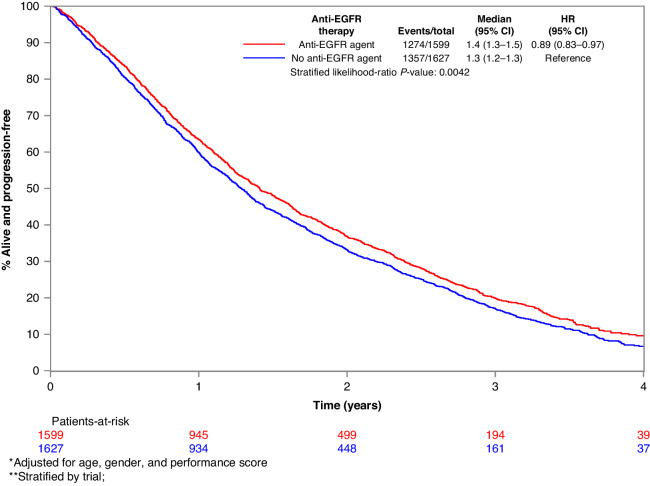
Kaplan–Meier PFS curves in the KRAS WT subgroup. Kaplan–Meier progression-free survival curves for KRAS wild-type subgroup of patients treated with EGFR mAb + chemo/BSC versus chemo/BSC alone. EGFR epidermal growth factor receptor.

EGFR mAb associated PFS benefit was larger in those with KRAS WT tumours if they were aged 70 or younger (HR_adj_ 0.68, *p* < 0.001) compared to those over 70 (HR_adj_ 0.93, *p* = 0.465). Other explored variables, including gender, site of metastatic disease, the extent of metastatic disease, previous resection of the bowel cancer primary, and the number of lines of therapy did not have a significant influence on the benefit associated with EGFR mAb in patients in KRAS WT mCRC. The forest plot of OS by variables of interest and the associated Kaplan–Meier survival curves for OS in the KRAS WT subgroup are depicted in Figs. 2 and 3, respectively. The forest plot of PFS according to the variables of interest, and the associated Kaplan–Meier PFS curves for PFS in the KRAS WT subgroup are depicted in Figs. 4 and 5, respectively.

**Fig. 6:**
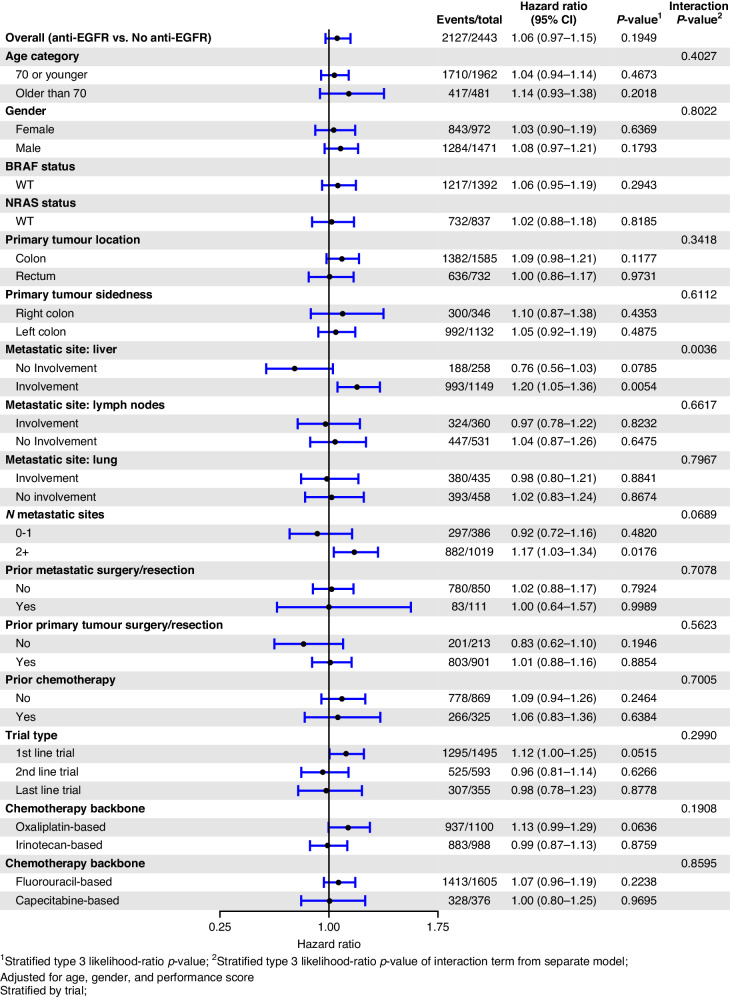
Forest plot—overall survival in the KRAS mutant subgroup. Forest plot of overall survival for KRAS mutant subgroup of patients treated with EGFR mAb + chemo/BSC versus chemo/BSC alone. EGFR epidermal growth factor receptor.

**Fig. 7:**
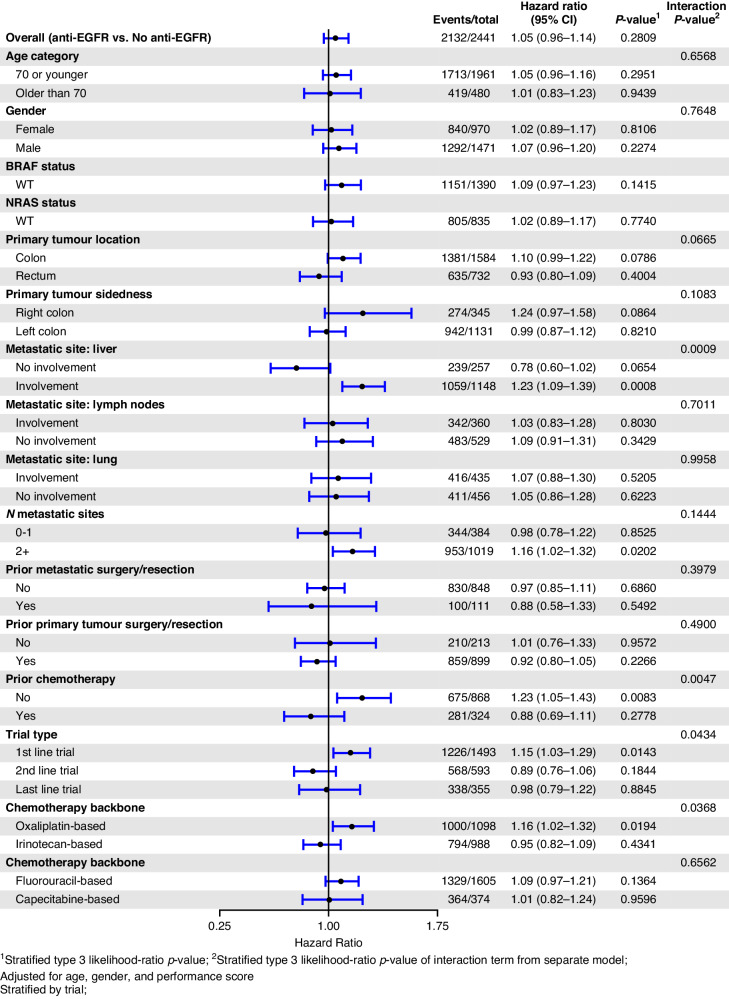
Forest plot—PFS in the KRAS MT subgroup. Forest plot of progression-free survival for KRAS mutant subgroup of patients treated with EGFR mAb + chemo/BSC versus chemo/BSC alone. EGFR epidermal growth factor receptor.

For the KRAS MT subgroup, the use of EGFR mAb did not improve OS (HR 1.06, 95% CI 0.97–1.15) or PFS (HR 1.05, 95% CI 0.96–1.14). Exploratory analyses showed a detrimental treatment effect of EGFR mAbs in KRAS mutant mCRC with liver metastasis (OS: HR_adj_ 1.20, *p* = 0.005, *p*_interaction_ = 0.004; PFS: HR_adj_ 1.23, *p* < 0.001, *p*_interaction_ < 0.001). In the KRAS MT cohort, the OS HR for those without liver metastases was 0.76 (*p* = 0.078) and PFS HR was 0.78 (p = 0.065). The forest plots of OS and PFS in the KRAS MT cohort according to the variables of interest are depicted in Figs. 6 and 7, respectively.

## Discussion

This is the largest IPD analysis to explore the predictive value of KRAS mutation status in mCRC. EGFR mAbs prolong OS and PFS in KRAS WT mCRC. KRAS mutation status has been adopted globally as a predictive biomarker for EGFR mAb efficacy [15]. Our findings support current clinical practice guidelines that restrict the use of EGFR mAbs to patients with mCRC which is KRAS WT. In contrast, those patients with mCRC that carry KRAS mutations do not benefit from EGFR mAb. We observed a lack of benefit using EGFR mAbs in NRAS mutant mCRC. Moreover, there was a trend toward inferior survival outcomes when EGFR mAb was used in the treatment of NRAS mutant mCRC. For BRAF mutant mCRC, we did not observe a benefit with EGFR mAb use, but the results did not suggest harm. In addition, the interaction test for BRAF mutation status was only significant for PFS and not OS. Further analysis of the impact of the BRAF status of mCRC, through later lines of therapy and by tumour sidedness, is warranted.

We explored multiple variables to identify potential patient subgroups within the KRAS MT cohort that may benefit from EGFR mAb therapy. We observed a non-statistically significant survival benefit for those patients without liver metastases. However, the use of EGFR mAbs was associated with a detrimental effect in patients with KRAS MT mCRC with liver involvement. These findings are intriguing and suggest that the pattern of cancer spread may influence the biological effects of anti-EGFR mAb therapy in the setting of KRAS MT mCRC, in either a positive or negative direction. These findings are best considered hypothesis-generating, especially as there is no treatment effect overall in the KRAS MT group.

Tumour-sidedness is a variable that has been incorporated into clinical practice, with left-sided location favoring EGFR mAb use compared to right-sided tumours [16, 17]. Our analysis supports this clinical practice, as we observed differences in EGFR mAb efficacy based on tumour sidedness. The survival benefit was statistically significant in the left-sided cancer cohort, but we could not demonstrate a benefit when patients with right-sided cancers received EGFR mAbs. When adding a monoclonal antibody to first-line chemotherapy, clinicians choose between bevacizumab and EGFR mAb. The side of cancer origin is an important factor in treatment decision consideration in current clinical practice. We did not consider studies that included bevacizumab in our meta-analysis.

The scale of our IPD analysis represents the largest dataset to examine a potential interaction between the type of chemotherapy and EGFR mAb effect. We observed a significant difference according to the type of fluoropyrimidine used. For patients with KRAS WT mCRC, EGFR mAb benefit was observed in those who received fluorouracil but there was no demonstrable benefit in those treated with capecitabine. A difference in EGFR mAb efficacy according to the type of fluoropyrimdine used in the chemotherapy backbone has been reported previously, with infusional fluorouracil associated with better outcomes when used with EGFR mAb than either capecitabine or bolus fluorouracil regimens [18, 19]. Differences in the toxicity profile of these chemotherapies plus EGFR mAb combinations, leading to dose reduction and treatment interruption, may explain these differences. Another purported biologically plausible mechanism is the ability of EGFR mAbs to reduce cell cycling through the promotion of G1 arrest. This reduces metabolic activation of capecitabine within cells leading to a reduced cytotoxic effect. It should be noted that only one study, the COIN study [13], used capecitabine in preference to fluorouracil in the chemotherapy doublet backbone. Another potential chemotherapy difference is the fluoropyrimdine doublet partnering drug, irinotecan versus oxaliplatin. Irinotecan-based chemotherapy regimens have been associated with greater EGFR mAb efficacy when compared to oxaliplatin-backbone regimens [18, 20]. We did not identify differences when comparing oxaliplatin-based chemotherapy regimens to regimens that use irinotecan.

Our results indicate a decline in benefit from EGFR mAb use with advancing age, specifically in those aged over 70. Reduced benefit with advanced age has been observed in previous studies of chemotherapy for mCRC. Potential reasons include increasing treatment toxicity with age, lower treatment dose delivery, and the effect of co-morbidities and competing risk factors for survival. Our findings highlight the need to consider the risks of toxicity and take into account co-morbidities when selecting the use of an EGFR mAb in patients aged over 70. One way of improving the safety profile of EGFR mAb-based therapy in the elderly is to consider reducing the intensiveness of the chemotherapy, using single-agent fluoropyrimidine instead of doublet chemotherapy, as demonstrated in the phase II PANDA study [21].

The meta-analysis supports the use of EGFR mAbs in the treatment of mCRC without mutations in KRAS, particularly for left-sided colorectal cancer and where the tumour does not harbour NRAS or BRAF mutations. The relative magnitude of benefit is greatest in later lines of therapy with single-agent EGFR mAb when compared to BSC. In earlier lines of therapy, when the EGFR mAb is combined with chemotherapy, fluorouracil-containing doublet chemotherapy regimens should be preferred to capecitabine-containing regimens. These findings could be considered in management guidelines to aid appropriate treatment decisions in clinical practice.

### Supplementary information

Supplemental Table 2

Supplemental Table 1

## Data Availability

The data sharing of individual patient data from each participating trial will be subject to the policy and procedures of the institutions and groups who conducted the original study.
