# Supplementary material for: Fluoropyrimidine type, patient age, tumour sidedness and mutation status as determinants of benefit in patients with metastatic colorectal cancer treated with EGFR monoclonal antibodies: individual patient data pooled analysis of randomised trials from the ARCAD database
Source: Br J Cancer. 2024 Feb 24;130(8):1269–78. doi: 10.1038/s41416-024-02604-y (PMC11015038; doi:10.1038/s41416-024-02604-y)

**Supplemental Table 2: Overall survival results from each included trial, according to KRAS status**


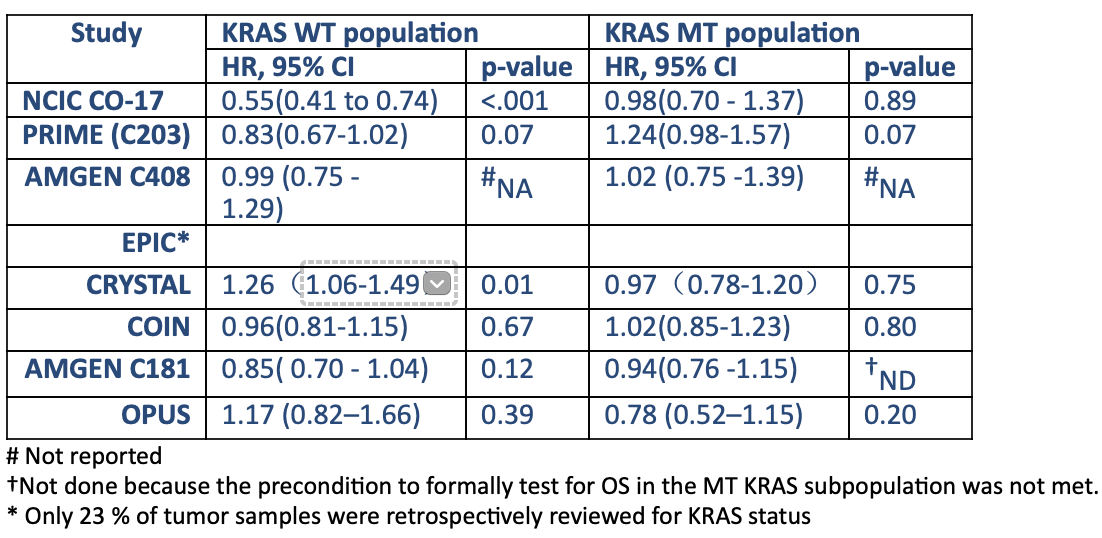

Supplement: Supplementary file 1 — Supplemental Table 2 [file 41416_2024_2604_MOESM1_ESM.docx]
