# Supplementary material for: Fluoropyrimidine type, patient age, tumour sidedness and mutation status as determinants of benefit in patients with metastatic colorectal cancer treated with EGFR monoclonal antibodies: individual patient data pooled analysis of randomised trials from the ARCAD database
Source: Br J Cancer. 2024 Feb 24;130(8):1269–78. doi: 10.1038/s41416-024-02604-y (PMC11015038; doi:10.1038/s41416-024-02604-y)
Supplement: Supplementary file 2 — Supplemental Table 1 [file 41416_2024_2604_MOESM2_ESM.docx]

**Supplement Table 1: Number of patients from the included randomised controlled studies**

| **Study Name** | **Years of Accrual** | **N Patients** | **Study Arm** | **N Patients in Arm with known KRAS Status** | **Trial Type** |
| --- | --- | --- | --- | --- | --- |
| **NCIC CO-17** | Dec 2003-Aug 2005 | 437 | Best Supportive Care + Cetuximab | 211 | Last Line Trial |
|  |  |  | Best Supportive Care | 204 |  |
| **PRIME (C203)** | Aug 2006-Feb 2008 | 866 | FOLFOX | 431 | First Line Trial |
|  |  |  | FOLFOX + Panitumumab | 435 |  |
| **AMGEN C408** | Jan 2004-Jun 2005 | 344 | Best Supportive Care | 175 | Last Line Trial |
|  |  |  | Best Supportive Care + Panitumumab | 169 |  |
| **EPIC** | May 2003-Feb 2006 | 300 | Irinotecan + Cetuximab | 146 | Second Line Trial |
|  |  |  | Irinotecan | 154 |  |
| **CRYSTAL** | Jul 2004-Nov 2005 | 1064 | FOLFIRI + Cetuximab | 530 | First Line Trial |
|  |  |  | FOLFIRI | 533 |  |
| **COIN** | Mar 2005-May 2008 | 1294 | Continuous FOLFOX | 205 | First Line Trial |
|  |  |  | Continuous CAPOX | 427 |  |
|  |  |  | Continuous FOLFOX + Cetuximab | 217 |  |
|  |  |  | Continuous CAPOX + Cetuximab | 434 |  |
| **AMGEN C181** | Jun 2006-Mar 2008 | 1083 | FOLFIRI + Panitumumab | 541 | Second Line Trial |
|  |  |  | FOLFIRI | 542 |  |
| **OPUS** | Jul 2005-Mar 2006 | 324 | FOLFOX + Cetuximab | 162 | First Line Trial |
|  |  |  | FOLFOX | 159 |  |
| **Total:** |  | 5712 |  | 5675 |  |
